# Neuroelectric and Behavioral Effects of Acute Exercise on Task Switching in Children with Attention-Deficit/Hyperactivity Disorder

**DOI:** 10.3389/fpsyg.2016.01589

**Published:** 2016-10-13

**Authors:** Chiao-Ling Hung, Chung-Ju Huang, Yu-Jung Tsai, Yu-Kai Chang, Tsung-Min Hung

**Affiliations:** ^1^Department of Athletic, National Taiwan UniversityTaipei, Taiwan; ^2^Graduate Institute of Sport Pedagogy, University of TaipeiTaipei, Taiwan; ^3^Department of Physical Education, National Taiwan Normal UniversityTaipei, Taiwan; ^4^Graduate Institute of Athletics and Coaching Science, National Taiwan Sport UniversityTaoyuan, Taiwan

**Keywords:** ERP, working memory, switch cost, executive function, physical activity

## Abstract

The main purpose of this two-part study was to examine the effects of acute, moderate intensity exercise on task switching in children with attention-deficit/hyperactivity disorder (ADHD). In Study 1, we compared the task switching performance of children with and without ADHD. Twenty children with ADHD and 20 matched controls performed the task switching paradigm, in which the behavioral indices and P3 component of event-related potentials elicited by task-switching were assessed simultaneously. The amplitude and latency of P3 reflected the amount of attention resource allocated to task-relevant stimulus in the environment and the efficiency of stimulus detection and evaluation, respectively. The task switching included two conditions; the pure condition required participants to perform the task on the same rule (e.g., AAAA or BBBB) whereas the mixed condition required participants to perform the task on two alternating rules (e.g., AABBAA…). The results indicated that children with ADHD had significantly longer RTs, less accuracy, and larger global switch cost for accuracy than controls. Additionally, ADHD participants showed smaller amplitudes and longer P3 latencies in global switch effects. In Study 2, we further examined the effects of an acute aerobic exercise session on task switching in children with ADHD. Thirty-four children with ADHD performed a task switching paradigm after 30 min of moderate-intensity aerobic exercise on a treadmill and after control sessions (watching videos while seated). The results revealed that following exercise, children with ADHD exhibited smaller global switch costs in RT compared with after control sessions. The P3 amplitude only increased following exercise in the mixed condition relative to the pure condition, whereas no effects were found in the control session. These findings suggest that single bouts of moderate intensity aerobic exercise may have positive effects on the working memory of children with ADHD.

## Introduction

Attention-deficit/hyperactivity disorder (ADHD) is one of the most common disorders in children, present in approximately 8.7% of children in the U.S. (Froehlich et al., 2007), and approximately 50% of children with ADHD experience symptoms that persist into adulthood (Lara et al., 2009). ADHD can cause numerous impairments in social, academic, and occupational functioning (Bledsoe et al., 2010), resulting in a substantial economic impact on society (Pelham et al., 2007). Medication, behavioral treatment, combinations of medication and behavioral treatment, and community care are four distinct treatment strategies for ADHD (Molina et al., 2009). Of these, the most widely and effectively used treatment for ADHD centers on pharmacotherapy, namely stimulants, as the first-line treatment in children with ADHD (Craig et al., 2015). Despite the positive effects of pharmaceutical treatment on the behavioral symptoms of ADHD, some potential side effects may occur, such as insomnia, appetite reduction, moodiness, and headaches (Schachter et al., 2001), and this type of treatment exhibits limited long-term gains that often disappear once treatment is discontinued (Chronis et al., 2006; Steinberg-Epstein et al., 2011). As a result, it is important to further investigate lifelong adjunctive or alternative potential treatment options that may benefit children with ADHD.

The executive function model (Pennington and Ozonoff, 1996) has been proposed to explain ADHD deficits (Sergeant et al., 2003). Emerging research consistently documents that ADHD is characterized by cognitive deficits, especially in executive function (Barkley, 1997; Barkley and Lombroso, 2000). Executive function (EF), or executive control, is an umbrella term that is used to describe “higher or meta-” cognitive function, including shifting, updating, inhibition (Miyake et al., 2000), switching, working memory, and sustained and selective attention (Alvarez and Emory, 2006). A previous meta-analytic study indicated that compared to healthy controls, children with ADHD consistently performed worse on EF tasks. The effect sizes of all measures fell within the medium range (0.46–0.69), but the strongest and most consistent effects were obtained for measures of response inhibition, vigilance, working memory, and planning (Willcutt et al., 2005). These studies point to a deficiency in multiple aspects of EF in ADHD.

Some studies have shown a positive effect of exercise or physical fitness on several aspects of EF in children with ADHD. For example, motor ability (Hung et al., 2013) and physical fitness (Tsai et al., 2016) were reported positively associated with better inhibitory function, a subcomponent of EF, in children with ADHD. Similarly, a long-term exercise program enhanced performance on inhibitory tasks (Chang et al., 2014) and idle state brain oscillations during a resting condition (Huang et al., 2014). Similarly, acute exercise benefits EF not only in healthy controls but also in children with ADHD. Medina et al. (2010) found that sustained attention was significantly improved following exercise. Furthermore, evidence indicated that acute exercise facilitated set shifting and inhibition performance as assessed by the Wisconsin Card Sorting Test and the Stroop Test, respectively (Chang et al., 2012). Similarly, Pontifex et al. (2012) found that single bouts of moderately intensive aerobic exercise improved inhibitory control in children with ADHD. The beneficial effects of acute exercise on inhibition was corroborated by a recent finding that acute exercise significantly improved performance on all three conditions of the Stroop Task but not on the Tower of London or Trail Making Test, in children with and without ADHD (Piepmeier et al., 2015).

In addition to behavioral indicators, event-related potentials (ERPs) have been used to assess the neurophysiological processes involved in the performance of executive function tasks. P3, one of the most studied ERP components, is a positive-going deflection in the ERP waveform that occurs in response to a stimulus, with the amplitude of the component reflecting the allocation of neural resources toward the stimulus (Polich, 2007). Therefore, measurement of P3 can reveal detailed neurophysiological information elicited by the EF task. In the only study involving acute exercise and EF performance with ERP measurement in children with ADHD, Pontifex et al. (2012) found that both children with ADHD and healthy controls exhibited larger P3 amplitudes during a flanker task, an inhibition task, after exercise compared with after reading, indicating acute exercise facilitate neurophysiological processes enabling regulatory adjustments in behavior. Thus, more studies employing ERPs to reveal the underlying neurophysiological processes for the benefit of acute exercise on the EF function in children with ADHD, are warranted.

Most of the studies examining the effects of acute exercise on EF in children with ADHD have focused on sustained attention (Medina et al., 2010), inhibitory control (Chang et al., 2012; Pontifex et al., 2012; Piepmeier et al., 2015), and set shifting (Chang et al., 2012). Whether the beneficial effects of exercise can be extended to other subcomponents of EF, working memory in particular, remains unknown. Task switching paradigms are one of the tasks that have been used extensively to examine the working memory, inhibition, and mental flexibility aspects of executive function (Monsell, 2003). The advantage of this paradigm is that it enables the separation of different components of executive control, such as task-set selection and maintenance (working memory), task-set switching (mental flexibility), and interference control (inhibition; Cepeda et al., 2001). A typical task switching paradigm consists of two conditions, such as repeated task trials in task-homogenous blocks (e.g., AAAA, BBBB: pure condition), switching trials in task-heterogeneous blocks (e.g., AB or BA: switching mixed condition), and non-switching or repeated task trials in task-heterogeneous blocks (e.g., AA or BB: non-switching mixed condition). The global switch costs, a measure of working memory, refer to the difference in reaction time (RT) between mixed and pure conditions, as this difference reflects the efficiency in maintaining multiple task sets in working memory as well as the selection of the task to be performed next (Kray and Lindenberger, 2000). The local switch costs, a measure of inhibition and mental flexibility, refer to the differences in reaction time between non-switching and switching trials in mixed condition, reflecting the effectiveness of the executive control processes responsible for activating the currently relevant task set and deactivating the task set that was relevant on the previous trial (Kray and Lindenberger, 2000).

Children with ADHD have shown impaired performance in task switching. Studies comparing task switching performance between children with and without ADHD have shown that children with ADHD demonstrate substantially larger switch costs than children without ADHD. However, when on medication, ADHD children’s switch performances were equivalent to those of control children. In addition, medication was observed to reduce ADHD children’s switch cost (Cepeda et al., 2000). Adults with ADHD also show generally slower and less accurate performances on task switching, suggesting deficits on several EF components in ADHD (King et al., 2007). Despite the lack of studies using ERPs to examine the differences on this topic, studies using functional magnetic resonance imaging (fMRI) have shown that ADHD adults do not display specific executive control problems at the behavioral level but do engage different brain areas during task switching than healthy controls; these differences include reduced activation in the bilateral inferior prefrontal cortex, caudate and thalamus during both Stop and Switching tasks, as well as in the left parietal lobe during the Switching task. The authors suggested that adults with childhood ADHD experienced reduced activation and inter-regional functional connectivity of fronto-striatal networks (Cubillo et al., 2010).

Based on the evidence mentioned above, acute exercise may be particularly beneficial for executive function. However, there are many different aspects of executive function. To our knowledge, no ERP studies to date have directly compared the differences between individuals with ADHD and healthy controls, and the effects of acute moderate exercise on the working memory, inhibition, and mental flexibility aspects of executive function in ADHD as measured by task switching have not yet been explored. Accordingly, we conducted two studies. In Study 1, we compared the task switching performance of children with and without ADHD to examine components of executive function deficiencies in task switching performance of children with ADHD. In Study 2, we further examined the effects of a single bout of acute aerobic exercise on components of executive function using the task switching performance of children with ADHD, particular regarding the aspects of working memory, inhibition, and mental flexibility. Based on previous findings, it was hypothesized that children with ADHD would show deficient task performance in task switching, along with reduced activation of the P3 component. Furthermore, acute exercise would improve performance during the task switching as well as demonstrate increased P3 amplitude and decreased P3 latency elicited by the task switching paradigm.

## Study 1

### Method

#### Participants

In total, 20 children with a clinical diagnosis of ADHD and 20 healthy match control children (all boys) were recruited through advertisements placed at a local elementary school and ADHD association. To be included in this study, participants had to meet the following criteria: (1) aged between 8 and 12 years (2) lack of hearing or vision problems and free of brain injury and disease (i.e., epileptic seizure) and (3) lack of comorbid developmental disorders including learning difficulties, dyslexia, Tourette’s syndrome, epilepsy and pervasive developmental disorder. All participants had not received medication for at least 24 h before the experiment. This study was conducted in accordance with the Declaration of Helsinki and approved by the National Taiwan University Institutional Review Board. Written assent was obtained from the children, and written informed consent was provided by their legal guardians. In addition to the formal diagnosis provide by a pediatrician or psychiatrist, their legal guardians confirmed the presence of the ADHD symptoms a priori by using the Chinese version of the ADHD test (Cheng, 2008) originally developed by Gilliam (1995), and the Chinese version of the Child Behavior Checklist (CBCL; Chen et al., 2006) originally developed by (Achenbach and Rescorla, 2001). All children were screened for IQ (Test of Non-verbal Intelligence) and were administered the Movement Assessment Battery for Children-2 (MABC-2).

#### Procedure

After receiving ethics approval, the participants visited the lab on two separate days. During the first visit, the experimental procedure was explained to participants and their legal guardians. Then a health history, demographics questionnaire, ADHD-T, CBCL, and an informed consent form were completed by their legal guardians. The participants were then requested to complete IQ and MABC-2. All of the participants’ heights and weights were also measured to calculate their body mass indexes (BMI).

At the second visit, the task switching experiment was explained and carried out in a sound and magnetic shielded room with dimmed lights. Before the formal experiment, the participants were outfitted with an electrode cap and instructed on the task. Both the pure and mixed condition trials were practiced until a criterion of 80% correction rate was reached by participants.

#### Task Switching Paradigm

Cognitive performance was assessed using the task switching paradigm modified from Dai et al. (2013), presented on a computer monitor controlled via Neuroscan Stim software (ver. 2.0; Neuro Inc., El Paso, TX, USA). In the task, a white numeric digit (digits 1–9, excluding 5) was presented in the center of a computer screen on a black background. The task include pure and mixed task conditions. Pure conditions include two subtests; for the first subtest, participants had to identify whether the number, surrounded by a solid-line rectangle, was smaller (1, 2, 3, and 4) or bigger (6, 7, 8, and 9) than 5 (i.e., AAA…). On the second subtest, the participants had to identify whether the number, surrounded by a dashed square, was odd (1, 3, 7, and 9) or even (2, 4, 6, and 8) (i.e., BBB…). For the mixed task condition, the two subtests from the pure task condition were combined to form an alternating-runs paradigm (i.e., AABBAA…) (Rogers and Monsell, 1995). The participants were instructed to press with their thumb of each hand to make a corresponding response on the key pad as quickly and accurately as possible. All eight digits appeared with equal probability in a random order. The digits were presented for 400 ms, with a 3000 ms response-stimulus interval. If no response was made, the trial was terminated 3500 ms after the onset of stimuli. Participants completed 64 trials in each of the pure task condition and 128 trials (64 trials × 2 blocks) in the mixed task condition. The first trial in each block for both pure task and mixed-task condition was discarded from the analyses. The viewing distance was approximately 60 cm and visual angles were 3.82°. The task performance measures for reaction time (RT), response accuracy, and global and local switch cost on RT were derived.

#### ERP Recording and Analysis

Event-related potential was measured with an electrode cap with electrodes placed at 30 sites using the 10–20 system; each electrode was referenced to an average of the mastoid electrodes, and the impedances were kept below 10 kΩ. Continuous data were digitized at a sampling rate of 500 Hz and amplified 500 times with a DC to a 70 Hz filter, and a 60-Hz notch filter was applied using a Neuroscan SynAmps2 amplifier. Only the ERP data recorded from the midline frontal (Fz), central (Cz), and parietal (Pz) locations were analyzed in this study (Dai et al., 2013). The offline data reduction included merging with the behavioral data. The ERP data were corrected for ocular artifacts. Epochs were defined as 100 ms pre-stimulus to 900 ms post-stimulus, and baseline corrections were performed using the 100-ms pre-stimulus interval. A low-pass filter with a 30 Hz cutoff (12 db/octave) was employed to further attenuate noise. ERP trials with amplitudes outside the range of ±100 μV were excluded from further analysis. The correct trials were separately averaged. To detect ERP components, P3 mean amplitudes were calculated for 300–700 ms time intervals within a 50-ms interval surrounding the largest positive going peak. Peak latencies were measured within the latency window.

#### Statistical Analysis

To ensure equivalence between the ADHD and control groups, a *t*-test was applied to compare the demographic data between the two groups. A 2 (Group: ADHD, Controls) × 2 (Condition: Pure and mixed) and a 2 (Group: ADHD, Controls) × 2 (Condition: non-switching and switching) mixed ANOVA were employed to analyze the RT and accuracy of the global switch and local switch, respectively. In addition, separate independent sample *t*-tests were used for global (the difference in RT and accuracy between the mixed and pure conditions) and local (the difference in RT and accuracy between non-switching and switching trials in the mixed condition) switch cost between the exercise and resting sessions. The P3 ERP component was assessed separately for amplitude and latency in 2 (Group: ADHD, Controls) × 2 (Condition: Pure and mixed or non-switching and switching trials) × 3 (site: Fz, Cz, Pz) mixed ANOVAs for global and local switch.

### Results

#### Demographic Analyses

There were no significant differences between the groups in age [*t*(38) = 0.09, *p* > 0.05], BMI [*t*(38) = 0.44, *p* > 0.05], IQ [*t*(38) = -1.88, *p* > 0.05], MABC-2 score [*t*(38) = -1.64, *p* > 0.05], suggesting equivalence between the two groups. However, as expected, the results indicated that children with ADHD scored significantly higher than the healthy controls on the ADHD-Q [*t*(38) = 7.41, *p* < 0.01] and CBCL [*t*(38) = 6.86, *p* < 0.01]. The demographic characteristics of participants in both groups are summarized in **Table 1**.

**Table 1:** Participant demographic characteristics for Study 1.

Variable	ADHD (*N* = 20)	Control (*N* = 20)	*P*
Gender (M:F; *M* [*SD*])	20:0	20:0	
Age (years; *M* [*SD*])	10.24 ± 1.78	10.20 ± 1.09	0.93
BMI (kg/m^2^; *M* [*SD*])	17.22 ± 4.14	16.77 ± 1.66	0.66
Test of Non-verbal	100.80 ± 15.28	108.25 ± 8.91	0.07
intelligence (score)		
MABC-2 (score)	10.55 ± 2.91	11.90 ± 2.25	0.11
ADHD Q (score)	97.05 ± 14.31	68.35 ± 9.76	<0.001
CBCL (score)	66.30 ± 7.39	46.25 ± 10.77	<0.001
ADHD subtype (*N* [%])			
ADHD-I	4 (20)		
ADHD-HI	1 (5)		
DAHD-C	15 (75)		
Medicine Intake	9 (45)		

*BMI, body mass index; ADHD Q, ADHD quotient, higher quotient indicates higher severity of the ADHD symptom; ADHD-I, predominantly inattentive subtype; ADHD-HI, predominantly hyperactive-impulsive subtype; ADHD-C, combined hyperactive-impulsive and inattentive subtype; *N*, number of participants; %, percentage in the group.*

#### Task Performance

##### Global switch

**Table 2** presents the detailed behavioral data for the task switching indices for each group. For reaction time, a two-way ANOVA revealed main effects of Group [*F*(1,38) = 10.97, *p* < 0.01, $\eta_{p}^{2}$ = 0.22] and Condition [*F*(1,38) = 140.74, *p* < 0.01, $\eta_{p}^{2}$ = 0.79], with the results indicating that children with ADHD responded slower than the controls. In addition, all participants responded faster during the pure condition compared to the mixed condition (see **Table 2**). The interaction of Group × Condition was not significant [*F*(1,38) = 0.06, *p* > 0.05].

**Table 2:** Means, and standard deviations for the task switching in Study 1.

Variable	ADHD (*n* = 20)	Control (*n* = 20)
	*M* (SD)	*M* (*SD*)
Global switch RT (ms)		
Pure trials	885.91 ± 139.13	691.42 ± 179.17
Mixed trials	1290.34 ± 247.89	1113.01 ± 248.23
Global switch cost	404.43 ± 261.15	421.59 ± 169.60
Local switch RT (ms)		
Non-switch trials	1255.39 ± 229.84	1066.36 ± 244.93
Switch trials	1324.98 ± 271.58	1163.96 ± 255.56
Local switch cost	69.59 ± 95.74	97.60 ± 74.76
Global switch accuracy (%)		
Pure trials	85.63 ± 10.05	92.14 ± 7.09
Mixed trials	69.67 ± 14.57	86.59 ± 10.65
Global switch cost	15.96 ± 11.11	5.56 ± 6.10
Local switch accuracy (%)		
Non-switch trials	70.24 ± 13.65	86.88 ± 10.72
Switch trials	69.12 ± 16.39	86.29 ± 11.56
Local switch cost	1.12 ± 7.76	0.59 ± 6.34

**FIGURE 1:**
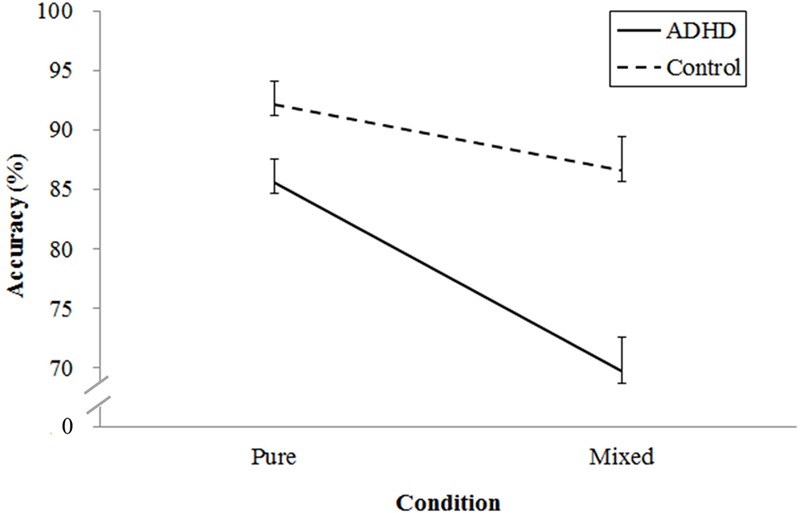
**Mean (±SE) response accuracy for pure and mixed conditions across ADHD and healthy control groups**.

For response accuracy, a two-way ANOVA revealed main effects of Group [*F*(1,38) = 13.99, *p* < 0.001, $\eta_{p}^{2}$ = 0.27] and Condition [*F*(1,38) = 54.76, *p* < 0.001, $\eta_{p}^{2}$ = 0.59], which were superseded by a Group × Condition interaction [*F*(1,38) = 12.80, *p* < 0.001, $\eta_{p}^{2}$ = 0.25]. Because there was a significant interaction effect, a follow-up of simple main effects analysis was utilized to decompose the Group × Condition interaction. A significant Condition effect was found for both groups, which revealed that ADHD [*F*(1,19) = 39.20, *p* < 0.001, $\eta_{p}^{2}$ = 0.67] and control [*F*(1,19) = 15.78, *p* < 0.001, $\eta_{p}^{2}$ = 0.45] groups showed higher response accuracy in the pure condition than in the mixed condition. Additionally, the significant group effect revealed that the ADHD group had lower accuracy than that of the controls in the pure [*F*(1,38) = 54.76, *p* < 0.001, $\eta_{p}^{2}$ = 0.59], and mixed condition [*F*(1,38) = 54.76, *p* < 0.001, $\eta_{p}^{2}$ = 0.59] (see **Figure 1**).

##### Local switch

Regarding reaction time, a two-way ANOVA revealed main effects for Group [*F*(1,38) = 5.01, *p* < 0.05, $\eta_{p}^{2}$ = 0.12] and Condition [*F*(1,38) = 37.89, *p* < 0.01, $\eta_{p}^{2}$ = 0.50], with the results indicating that controls responded faster than participants with ADHD. In addition, all participants responded faster during the non-switch condition compared to the switch condition (**Table 2**). The interaction of Group × Condition was not significant [*F*(1,38) = 1.06, *p* > 0.05].

For response accuracy, although no main effects were observed for Condition [*F*(1,38) = 0.55, *p* > 0.05] or the interaction of Group × Condition [*F*(1,38) = 0.05, *p* > 0.05], a significant main effect of Group [*F*(1,38) = 17.56, *p* < 0.01, $\eta_{p}^{2}$ = 0.32] was shown, with higher accuracy in the control than in the ADHD group (**Table 2**).

##### Global and local switch costs

There were no significant differences between groups in the global switch costs [*t*(38) = -0.25, *p* > 0.05] or local switch costs [*t*(38) = -1.03, *p* > 0.05] for the reaction time. However, a global switch cost for accuracy was observed [*t*(38) = 3.58, *p* < 0.001]. Examination of the means indicated that the global switch cost in ADHD participants (*M* = 15.9%, *SE* = 2.55) was significantly higher than that in controls (*M* = 5.56, *SE* = 1.40). There was no group difference in local switch cost for accuracy [*t*(38) = 0.23, *p* > 0.05].

#### ERP Data

##### Global switch: P3 amplitudes

**FIGURE 2:**
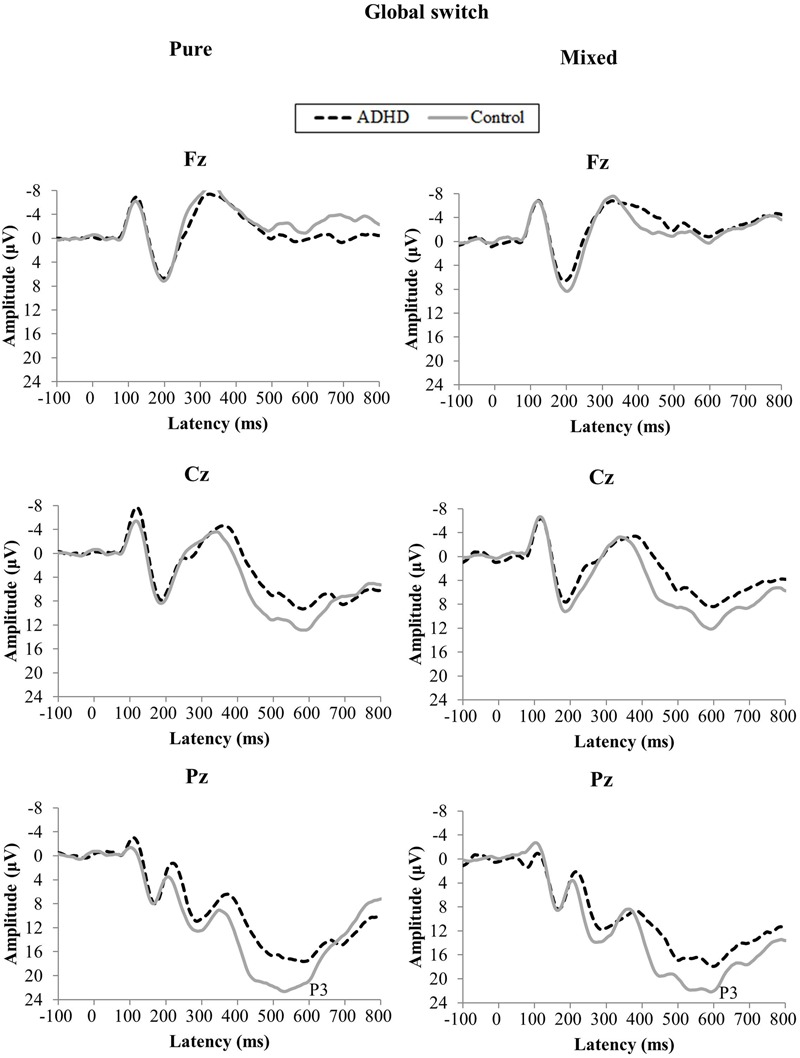
**Grand average event-related potentials (ERPs) for pure and mixed conditions of global switch at the Fz, Cz, and Pz sites stratified by group**.

The grand average ERP waveforms for task switching, group, and site are illustrated in **Figure 2**. A three-way ANOVA revealed main effects of Group [*F*(1,38) = 13.99, *p* < 0.001, $\eta_{p}^{2}$ = 0.27] and Condition [*F*(1,38) = 54.76, *p* < 0.001, $\eta_{p}^{2}$ = 0.59], which were superseded by a Group by Site interaction [*F*(2,76) = 4.38, *p* < 0.05, $\eta_{p}^{2}$ = 0.10], but no effects were observed for the three-way or other two-way interactions and main effects. Decomposition of the interaction between Group and Site revealed a significant Group effect in the Pz [*t*(19) = -2.11, *p* < 0.05], with a greater amplitude in controls (*M* = 19.98 μV, *SE* = 1.28) than in participants with ADHD (*M* = 15.85 μV, *SE* = 1.48). Furthermore, a significant Site effect in the ADHD group was revealed [*F*(1,38) = 145.75, *p* < 0.01, $\eta_{p}^{2}$ = 0.88], with greater amplitudes in the following order: Pz (*M* = 15.85 μV, *SE* = 1.48) > Cz (*M* = 6.09 μV, *SE* = 1.60) > Fz (*M* = -2.21 μV, *SE* = 1.43). For controls, the same effects were found [*F*(1,38) = 144.06, *p* < 0.01, $\eta_{p}^{2}$ = 0.88], with greater amplitudes in the following order: Pz (*M* = 19.98 μV, *SE* = 1.28) > Cz (*M* = 9.49 μV, *SE* = 0.78) > Fz (*M* = -1.25 μV, *SE* = 0.87).

##### Global switch: P3 latencies

A three-way ANOVA revealed an interaction of Group and Condition [*F*(1,38) = 5.29, *p* < 0.05, $\eta_{p}^{2}$ = 0.12], but no effects were observed for the three-way or other two-way interactions and main effects. Decomposition of the interaction between Group and Condition revealed a significant Group effect in pure trials [*t*(38) = 3.21, *p* < 0.01], with a shorter latency in controls (*M* = 541.67, *SE* = 10.71) than in ADHD participants (*M* = 592.23, *SE* = 11.55). However, there were no significant effects for mixed trials. Furthermore, a significant Condition effect in controls [*t*(19) = -2.38, *p* < 0.05] was observed, with a shorter latency in pure trials (*M* = 541.67, *SE* = 10.71) than in mixed trials (*M* = 577.70, *SE* = 12.75). No effects were found in the ADHD group.

##### Local switch: P3 amplitudes and latency

No three- or two-way interactions or main effects were observed in P3 amplitudes and latency.

## Study 2

### Method

#### Participants

In this study, 36 children with a clinical diagnosis of ADHD were recruited through advertisements placed at a local elementary school. The inclusion criteria were the same as those of Study 1. Two participants were excluded from the ERP analysis due to contaminated (e.g., VEOG, HEOG, and electromyogram) ERP data. All participants met the criteria assessed by the Physical Activity Readiness Questionnaire (PAR-Q) before performing the single bout of aerobic exercise to ensure no potential risk factors while performing a single bout of aerobic exercise.

#### Procedure

**Day 1.** In the first day, the participants and their parents completed all of the paperwork and tests as described in Study 1.

**Days 2 and 3.** During the second and third laboratory visits, participants visited the laboratory at the same time of day on two separate days within a given week. After arriving at the lab, the participants were connected to a HR monitor and had their HR recorded after 5 min of seated rest. The participants were counterbalanced into a resting and an exercise session, to minimize the potential order effect. In the resting session, the participants watched a video for 30 min. In the exercise session, the participants were instructed to complete a 30 min treadmill exercise. Participants were equipped with a Neuroscan Quickcap and administered cognitive tasks following each resting/exercise session. The task switching paradigm and neuroelectrical measurements were the same as those in Study 1.

#### Acute Exercise Session Manipulation

In the moderate-intensity aerobic exercise session, the participants were instructed to complete 30 min of treadmill exercise, including 5 min of warming up, 20 min of main exercise, and 5 min of cooling down (Chang et al., 2012). The exercise intensity was measured by heart rate reserve (HRR), which was calculated by the formula of Karvonen et al. (1957) as maximal HR minus resting HR, and maximal HR was estimated with an indirect formula of “206.9 – (0.67 × age)” (Gellish et al., 2007). The target HR was calculated by a formula as follows: Target HR = [(maximal HR – resting HR) × percentage intensity desired + resting HR]. Moderate intensity was set at 50–70% HRR for each participant’s individual HRR. Heart rate (HR) was measured with a Polar heart monitor (Polar RS800CX; Polar Electro Oy, Kempele, Finland) throughout the test. Treadmill velocity was slightly modified based on HR. Three HR data points were recorded, including resting HR, post-exercise HR and mean HR. In addition, ratings of perceived exertion (RPE; Borg, 1998), which provided a subjective rating of each individual’s perceptions of effort during exercise, were assessed every 2 min. We utilized the modify Borg scale, which ranges from 0 to 10 scores.

#### Statistical Analysis

To assess the exercise intensity manipulation, a 2 (Session: exercise, resting) × 3 (Time: pre-HR, avg-HR, and post-HR) repeated measures ANOVA for HR was performed.

A 2 (Session: post-exercise, post-resting) × 2 (Condition: Pure and mixed) and a 2 (Session: post-exercise, post-resting) × 2 (Condition: non-switching and switching trials in the mixed condition) repeated measures ANOVA were employed to analyze the RT and accuracy of the global switch and local switch, respectively. In addition, separate paired t-tests were used for global (the difference in RT and accuracy between mixed and pure conditions) and local (the difference in RT and accuracy between non-switching and switching trials in the mixed condition) switch cost between the exercise and resting sessions.

The P3 ERP component was assessed separately for amplitude and latency in 2 Session: post-exercise, post-resting) × 2 (Condition: pure and mixed or non-switch and switch trials) × 3 (site: Fz, Cz, Pz) analyses for global and local switch.

### Results

#### Demographic Information

The demographic characteristics of participants in Study 2 are summarized in **Table 3**.

**Table 3:** Participant demographic characteristics for Study 2.

Variable	ADHD (*N* = 34)
Gender (M:F; *M* [*SD*])	33:1
Age (years; *M* [*SD*])	10.16 ± 1.74
BMI (kg/m^2^; *M* [*SD*])	17.81 ± 3.80
Test of Non-verbal Intelligence (score)	104.91 ± 16.89
MABC-2 (score)	10.68 ± 2.90
ADHD Q (score)	98.66 ± 14.65
CBCL (score)	67.53 ± 7.55
ADHD subtype (*N* [%])	
ADHD-I	8 (23.5)
ADHD-HI	2 (5.9)
ADHD-C	24 (70.6)
Medicine Intake	14 (41.2)

*ADHD-I, predominantly inattentive subtype; ADHD-HI, predominantly hyperactive-impulsive subtype; ADHD-C, combined hyperactive-impulsive and inattentive subtype; *N*, number of participants; %, percentage in the group.*

#### Exercise Manipulation Check

The descriptive data for HR and RPE are summarized in **Table 4**. The results of the 2 × 3 repeated ANOVA for HR revealed a significant main effects of Session [*F*(1,33) = 889.7, *p* < 0.001, $\eta_{p}^{2}$ = 0.96] and Time [*F*(2,66) = 802.66, *p* < 0.001, $\eta_{p}^{2}$ = 0.96], which were superseded by a Session × Time interaction [*F*(2,66) = 988.88, *p* < 0.01, $\eta_{p}^{2}$ = 0.97]. The follow-up simple main effects analysis revealed a significant session effect for avg-HR [*t*(33) = 66.94, *p* < 0.01] and post-HR [*t*(33) = 8.75, *p* < 0.01], but not pre-HR [*t*(33) = 0.25, *p* > 0.05]. Examination of the means showed that the avg-HR and post-HR were significantly higher for the exercise than for the resting session.

**Table 4:** Descriptive data for exercise manipulation check in Study 2.

Variable	Control	Exercise
	*M* (*SD*)	*M* (*SD*)
HR-pre (bpm)	83.88 ± 8.30	83.62 ± 7.26
HR-avg. (bpm)	155.11 ± 3.41	81.5 ± 6.81
HR-post (bpm)	97.82 ± 11.34	80.91 ± 7.03
RPE-avg.	-	5.08 ± 1.58

*bpm, beats per minute; -pre, variable assessed before each treatment interventions; -avg., Average variable assessed during the treatment stage; -post, variable assessed immediately before cognitive task.*

#### Task Performance

##### Global switch

**Table 5** presents the detailed behavioral data for the task switching indices for each group. For reaction time, the results of the 2 × 2 repeated ANOVA revealed no significant main effects of Session [*F*(1,33) = 0.22, *p* > 0.05], but significant main effects of Condition [*F*(1,33) = 106.01, *p* < 0.001, $\eta_{p}^{2}$ = 0.76], which were superseded by a significant Session × Condition interaction [*F*(1,38) = 12.80, *p* < 0.01, $\eta_{p}^{2}$ = 0.25]. The follow-up simple main effects analysis revealed significant condition effects for both sessions. Participants responded faster in the pure condition than in the mixed condition both post-exercise [*t*(33) = 8.94, *p* < 0.01] and post-resting [*t*(33) = -9.31, *p* < 0.01]. However, no significant differences in pure [*t*(33) = 1.85, *p* > 0.05] or mixed [*t* (33) = -0.95, *p* > 0.05] conditions were observed between the two types of sessions.

**Table 5:** Means and standard deviations for the task switching in Study 2.

Variable	Post-exercise	Post-resting
	*M* (*SD*)	*M* (*SD*)
Global switch RT (ms)		
Pure trials	951.96 ± 193.01	900.93 ± 172.54
Mixed trials	1244.95 ± 258.75	1274.02 ± 252.89
Global switch cost	292.99 ± 191.13	373.09 ± 233.71
Local switch RT (ms)		
Non-switch trials	1205.29 ± 254.45	1237.14 ± 245.97
Switch trials	1287.36 ± 280.19	1311.49 ± 269.15
Local switch cost	82.07 ± 132.36	74.34 ± 111.23
Global switch accuracy (%)		
Pure trials	85.24 ± 9.40	87.29 ± 8.86
Mixed trials	73.32 ± 13.49	73.06 ± 14.58
Global switch cost	11.92 ± 11.34	14.23 ± 10.68
Local switch accuracy (%)		
Non-switch trials	74.45 ± 13.05	73.21 ± 14.62
Switch trials	72.21 ± 14.72	72.92 ± 15.72
Local switch cost	2.24 ± 6.90	0.29 ± 8.52

For response accuracy, although no main effects were observed for Session [*F*(1,33) = 0.41, *p* > 0.05] or for the interaction of Session × Condition [*F*(1,33) = 1.36, *p* > 0.05], a significant main effect of Condition [*F*(1,33) = 65.98, *p* < 0.01, $\eta_{p}^{2}$ = 0.67] was shown, with more accuracy in the pure (*M* = 86.27%, *SE* = 1.19) than in the mixed (*M* = 73.19%, *SE* = 2.32) condition.

##### Local switch

For reaction time, a 2 × 2 mixed ANOVA revealed that there was a significant main effect of Condition [*F*(1,33) = 19.79, *p* < 0.01, $\eta_{p}^{2}$ = 0.37]; participants responded faster in the non-switching (*M* = 1221.22 ms, *SE* = 40.41) than in the switching (*M* = 1299.42 ms, *SE* = 43.54) trials. However, the main effects of Session [*F*(1,33) = 0.84, *p* > 0.05] and the interaction of Session × Condition [*F*(1,33) = 0.11, *p* > 0.05] were not significant.

For response accuracy, no effects were observed for the main effect of Session [*F*(1,33) = 0.04, *p* > 0.05] or Condition [*F*(1,33) = 2.04, *p* > 0.05] or the interaction of Session × Condition [*F*(1,33) = 0.97, *p* > 0.05].

##### Global and local switch costs

As for RT, there were significant differences in global switch costs [*t*(33) = -2.34, *p* < 0.05] between post-resting (*M* = 373.09 ms, *SE* = 40.08) and post-exercise sessions (*M* = 292.99 ms, *SE* = 32.78). However, no significant differences in local switch costs [*t*(38) = -1.03, *p* > 0.05] were observed.

With regards to response accuracy, no significant differences in global [*t*(33) = -1.17, *p* > 0.05] and local switch costs [*t*(33) = 0.98, *p* > 0.05] were observed.

#### ERP Data

##### Global switch: P3 amplitudes and latency

**FIGURE 3:**
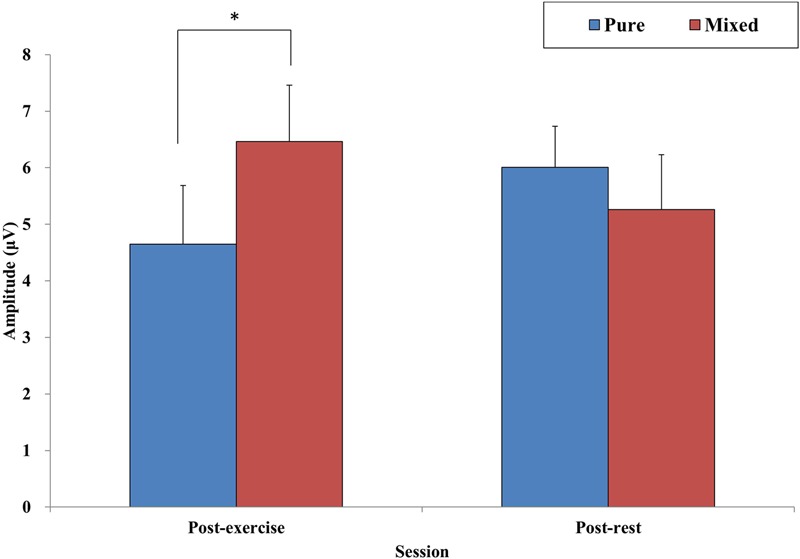
**Mean amplitude of three sites (Fz, Cz, and Pz) in pure and mixed conditions for post-exercise and post-resting session.** ^∗^*p* < 0.05.

A three-way ANOVA revealed an interaction of Session and Condition [*F*(1,33) = 8.67, *p* < 0.01, $\eta_{p}^{2}$ = 0.21], but no effects were observed for the three-way or other two-way interactions or for the main effects. A decomposition of the interaction between Session and Condition revealed a significant Condition effect in the post-exercise session [*t*(33) = -2.32, *p* < 0.05], with a greater amplitude in mixed trials (*M* = 6.46 μV, *SE* = 0.72) than in pure trials (*M* = 4.65 μV, *SE* = 1.04). However, no effects were found in the post-resting session (see **Figure 3**). Regarding P3 latency, no three- or two-way ANOVA interactions or main effects were observed.

##### Local switch: P3 amplitudes and latency

No three- or two-way ANOVA interactions or main effects were observed in P3 amplitudes and latency.

## Discussion

The purposes of this two-part study were to examine the differences in task switching performance in children with and without ADHD and to examine the effects of acute exercise on the switching aspect of executive function, particularly regarding the aspects of working memory, inhibition, and mental flexibility in children with ADHD. In Study 1, the results indicated that children with ADHD demonstrated significantly longer RTs and less accuracy during global switch and local switch conditions than controls. Moreover, children with ADHD had higher global switch cost for accuracy. The ERPs of ADHD participants showed smaller amplitudes and longer latencies in P3 in global switch effects. As for Study 2, the results revealed that following a single 30-min bout of exercise, children with ADHD exhibited smaller global switch costs on RT compared with the costs after a control session. The P3 amplitude only increased following exercise in mixed conditions relative to the pure condition, whereas no effects were found in the control session.

The slower RT in both task switch condition and higher global switch cost for accuracy in children with ADHD are consistent with those of Wu et al. (2006), who found generally slower response times and less accurate responses in a task switching paradigm in children with ADHD. These deficiencies may persist into adulthood, as King et al. (2007) also observed a generally slower and less accurate performance in task switching in adults with ADHD. Global switch costs have been linked to the ability to maintain and schedule multiple task sets and working memory that responds to loading multiple tasks (Kray and Lindenberger, 2000). The finding of higher global switch cost in the present study suggests deficits in working memory in the children with ADHD. This deficiency has been observed in adults with ADHD as well (White and Shah, 2006). This suggests that both children and adults with ADHD may be specifically impaired in working memory, a result consistent with previous findings suggesting working memory is one of the EF components which showed stronger and consistent deficiency in ADHD participants (Willcutt et al., 2005).

The smaller P3 amplitudes in global switch effects in children with ADHD suggest deficiencies in neural resource allocation when challenged with demands on inhibition, mental flexibility, and working memory. Despite the limited research on task switching using ERPs in ADHD participants, the available studies on different executive function tasks also demonstrated smaller P3 amplitudes in ADHD participants. Specifically, during a continuous performance test, a task that demands sustained attention, children with ADHD-com showed smaller frontal N1 and N2 amplitudes and parietal P2 and P3 amplitudes to target stimuli, indicating diminished evaluative and processing capabilities (Lawrence et al., 2005). Similarly, children with ADHD-com exhibited smaller N2 and P3 amplitudes to incongruent flankers, suggesting problems in conflict/inhibition processing (Johnstone et al., 2009).

Additionally, compared to healthy controls, children with ADHD demonstrated a longer P3 latency in the pure condition. Furthermore, only healthy controls showed differences in P3 latency between pure and mixed conditions. This longer P3 latency during task switching in ADHD participants was also observed in other tasks involving executive function. For example, Fisher et al. (2011) found that ADHD participants not only made significantly more commission errors than controls on NoGo trials but also exhibited longer N2 and P3 latencies on a Go/Nogo task. They proposed that the core deficit of ADHD pertained to a regulation disorder that could manifest in a lack of inhibition (commissions) in some situations or over-inhibition in others (omissions), depending on the circumstances. Notably, our ADHD participants showed longer P3 latencies in the pure condition, a condition that requires less mental effort, suggesting an inefficient and possibly disordered regulation in this population. Taken together, our results suggest an impaired working memory at the behavioral level and a slower cognitive processing and classification speed with regulation disorder in children with ADHD.

Although children with ADHD have shown deficiency in working memory as reflected in higher global switch cost, acute exercise can improve working memory. Specifically, compared with the control session, the acute exercise resulted in smaller global switch costs in RT in children with ADHD. The selective effect of acute exercise on global switch cost is consistent with findings regarding chronic exercise in elderly participants. Themanson et al. (2006) found a lower global switch cost in older adults with higher levels of physical activity than the lower levels of physical activity counterparts. Similarly, older participants who regularly engage in open-skill exercise demonstrated lower global switch costs than sedentary controls (Dai et al., 2013). These results suggest that physical activity may exhibit a greater influence on global switch costs due to the increased working memory load required in mixed trials compared to pure trials (Themanson et al., 2006). Regarding acute exercise and individuals with ADHD, previous studies have shown benefits of acute exercise on other aspects of executive function using the Stroop task (Chang et al., 2012; Piepmeier et al., 2015), Wisconsin Card Sorting Test (Chang et al., 2012), FLanker task (Pontifex et al., 2012), and Go/Nogo tasks (Chuang et al., 2015). Additionally, a meta-analytical review has shown that acute, intermediate-intensity exercise has a strong beneficial effect on response speed in working memory tasks (McMorris et al., 2011). This is the first study to demonstrate that the benefits of acute exercise can be extended to global switch cost in ADHD and suggests that working memory in children with ADHD is more susceptible to the effects of acute exercise. This finding may be particularly important because children with ADHD frequently exhibit impairments in working memory (Martinussen et al., 2005; Willcutt et al., 2005).

Enhanced P3 amplitudes in the mixed condition relative to the pure condition after acute exercise suggests enhancement of working memory processes and is consistent with the behavioral results. A previous study using a flanker task found that following acute exercise, both children with ADHD and healthy controls exhibited larger P3 amplitudes (Pontifex et al., 2012). Similarly, acute exercise also enhanced the preparatory process measured by contingent negative variation (CNV) in children with ADHD (Chuang et al., 2015). These findings of enhanced regulatory processes following acute exercise are particularly beneficial for children with ADHD, considering the fact that one of the core deficits of ADHD is disordered regulation (Fisher et al., 2011).

Although the mechanisms behind the beneficial effects of acute exercise on task switching in children with ADHD have yet to be fully understood, the facilitation effects of acute exercise may potentially be explained by changes in arousal. As the hypoarousal model of ADHD (Satterfield and Cantwell, 1974) suggests, children with ADHD are characterized by a generally lower arousal, and thus acute bouts of exercise that generally arouse the organism can lead to changes in cognitive function (Kamijo et al., 2004). The upregulation of neurochemicals such as neurotrophins (e.g., brain-derived neurotrophic factor, BDNF) and neurotransmitters (e.g., dopamine and norepinephrine) is another possible explanation. Dopamine would be the most likely candidate for the beneficial effects of acute exercise on global switch cost because of its positive effects on processing speed in tasks involving working memory (McMorris et al., 2011). Using spontaneous eye blinks and the acoustic startle eye blink response as non-invasive measures of dopamine, Tantillo et al. (2002) showed the effectiveness of acute exercise on increasing dopamine levels in children with ADHD. This dopamine hypothesis is also consistent with stimulant treatment, such as with MPH, which increases dopamine levels by blocking dopamine transporters (Swanson and Volkow, 2003) and improves performance on aspects of executive function including spatial working memory, response inhibition, and set-shifting (Arnsten, 2006).

It is important to note that although this study provides evidence supporting the beneficial effects of acute exercise on task switching in children with ADHD using behavioral and neuroelectric measures, several issues should be considered for future research efforts. First, the severity of ADHD symptoms was not evaluated in this study. However, given that 41% of the children in the present study were not receiving medication, it is likely that the participants had mild to moderately severe ADHD symptoms. Therefore, the extent to which we can generalize our results to individuals with more severe ADHD symptoms is unknown. Thus, future research should characterize ADHD symptoms to better understand the utility of acute exercise in enhancing switching ability in these populations. Second, issues related to exercise intensity, types of exercise modality, dose–response relationships, potential moderators (i.e., physical fitness), and time delay effects (Barella et al., 2010), need to be considered in future efforts to design better exercise regimens for adjunctive treatment of children with ADHD.

## Conclusion

The present study extends current knowledge on the relationship between acute exercise and executive function in children with ADHD. This study showed that children with ADHD experience certain deficiencies in performing task switching compared to healthy controls. Additionally, the findings indicated that acute bouts of exercise can ameliorate these deficiencies, especially those regarding switching conditions that require greater working memory involvement.

## Author Contributions

CLH is responsible for the research idea, implementing the study and manuscript writing up. CJH is responsible for the statistical support and discussion commentary. YJT is responsible for assisting on the data collection and analysis. YKC is responsible for consulting the methodology and interpretation of the findings. TMH is responsible for the discussion of the research idea, supervision of the data collection, and comment on the manuscript writing up.

## Conflict of Interest Statement

The authors declare that the research was conducted in the absence of any commercial or financial relationships that could be construed as a potential conflict of interest.
